# Punished for Substitution

**Published:** 1899-12

**Authors:** 


### Punished for Substitution.

A decision of considerable importance was made by Judge Kohlsaat in the United States Circuit Court yesterday. In the bill for an injunction, Fairchild Brothers & Foster of New York, had charged Edward Otto, a Chicago druggist, with substituting a spurious and inferior preparation for "Fairchild's Essence of Pepsine" in several cases where the latter was expressly called for in physicians' prescriptions. The case was hotly contested, and hundreds of pages of depositions were taken in New York and Chicago. Judge Kohlsaat's decree sustains the charges made, perpetually enjoins Otto from ever repeating the offense, and taxes him with the costs, amounting to about $500. This is said to be the first contested case in the United States in which the principle of protection to trade marks and trade names was extended so as to apply to what is technically known in the drug business as "substitution." Judge Kohlsaat's decision will probably protect manufacturing chemists, physicians and the general public, all of whom have in the past suffered from these fraudulent practices of a certain class of druggists.—*Chicago Times-Herald*, Friday, October 13, 1899.

